# Disruption of stomatal lineage signaling or transcriptional regulators has differential effects on mesophyll development, but maintains coordination of gas exchange

**DOI:** 10.1111/nph.14746

**Published:** 2017-08-21

**Authors:** Graham J. Dow, Joseph A. Berry, Dominique C. Bergmann

**Affiliations:** ^1^ Department of Biology Boston University Boston MA 02215 USA; ^2^ Department of Global Ecology Carnegie Institution for Science Stanford CA 94305 USA; ^3^ Department of Biology Stanford University Stanford CA 94305 USA; ^4^ HHMI Stanford University Stanford CA 94305 USA

**Keywords:** *Arabidopsis thaliana*, gas‐exchange capacity (*g*_smax_), leaf photosynthetic potential (*V*_cmax_), mesophyll development, stomatal development, water‐use efficiency

## Abstract

- Stomata are simultaneously tasked with permitting the uptake of carbon dioxide for photosynthesis while limiting water loss from the plant. This process is mainly regulated by guard cell control of the stomatal aperture, but recent advancements have highlighted the importance of several genes that control stomatal development.
- Using targeted genetic manipulations of the stomatal lineage and a combination of gas exchange and microscopy techniques, we show that changes in stomatal development of the epidermal layer lead to coupled changes in the underlying mesophyll tissues. This coordinated response tends to match leaf photosynthetic potential (*V*_cmax_) with gas‐exchange capacity (*g*_smax_), and hence the uptake of carbon dioxide for water lost.
- We found that different genetic regulators systematically altered tissue coordination in separate ways: the transcription factor SPEECHLESS (SPCH) primarily affected leaf size and thickness, whereas peptides in the EPIDERMAL PATTERNING FACTOR (EPF) family altered cell density in the mesophyll. It was also determined that interlayer coordination required the cell‐surface receptor TOO MANY MOUTHS (TMM).
- These results demonstrate that stomata‐specific regulators can alter mesophyll properties, which provides insight into how molecular pathways can organize leaf tissues to coordinate gas exchange and suggests new strategies for improving plant water‐use efficiency.

## Introduction

Recent attention has turned to the contributions of stomatal development in optimizing plant–environment relationships and controlling physiological performance (Chater *et al*., 2014; Dow & Bergmann, 2014; Lawson & Blatt, 2014). Enabling this focus is the availability of genetic resources to modify stomatal numbers and their distribution, or pattern, on the leaf surface (Lau & Bergmann, 2012; Pillitteri & Torii, 2012). Several groups have analyzed mutants or transgenic lines that specifically altered stomatal lineage transcription factors or signaling components with traditional physiological and environmental tools (Doheny‐Adams *et al*., 2012; Tanaka *et al*., 2013; Dow *et al*., 2014b; Franks *et al*., 2015). Gas‐exchange experiments in *Arabidopsis thaliana* identified a connection between *g*_smax_, the anatomical maximum rate of stomatal conductance as defined by stomatal size and density, and photosynthetic rate (Dow *et al*., 2014a,b). When *g*_smax_ was substituted for net carbon assimilation (*A*) in the Ball–Berry equation (Ball *et al*., 1987), this derived model was capable of predicting operational stomatal conductance. The ability to substitute *g*_smax_ for *A* hinted at an underlying link between stomatal development and the photosynthetic potential of the leaf**.** Here, we investigate the impact of genetic manipulations in the stomatal lineage on the developmental organization and physiological capacity of the mesophyll tissue.

## Materials and Methods

### Plant materials and growth conditions

All genotypes tested were in the Columbia (Col‐0) ecotype of *Arabidopsis thaliana* (L.) Heynh. and Col‐0 was used as the control in all experiments. The following previously described genotypes were used: *epf1* and *tmm;epf1* (Hara *et al*., 2007); *epf2*,* epf1;epf2*, and *tmm;epf2* (Hunt & Gray, 2009); 35S_pro_:EPF1 and 35S_pro_:EPF2 (Hara *et al*., 2009); SPCH_pro_:SPCH‐YFP and SPCH_pro_:SPCH 2‐4A‐YFP (Lampard *et al*., 2008); SPCH SILENCE (Dow *et al*., 2014b); *basl* (Dong *et al*., 2009); *tmm‐1* (Nadeau & Sack, 2002); *erecta‐105* (Torii *et al*., 1996); *tmm;erecta* (Shpak *et al*., 2005); 35S_pro_:EPFL9OX and 35S_pro_:EPFL9RNAi, referenced in this manuscript as STOMAGEN OX and STOMAGEN RNAi, respectively (Hunt *et al*., 2010). Seeds were surface‐sterilized and stratified at 4°C for 3–5 d in 0.15% agarose solution and then sown directly into Pro‐Mix HP soil (Premier Horticulture; Quakerstown, PA, USA) and supplemented with Scott's Osmocote Classic 14‐14‐14 fertilizer (Scotts‐Sierra, Marysville, OH, USA). At 10–14 d, seedlings were thinned so only one seedling per container remained. Plants were grown to maturity in growth chambers where the conditions were as follows: 16 : 8 h, 22 : 20°C, day : night cycle, *c*. 100 μmol photon m^−2^ s^−1^, unless otherwise noted.

### Calculating *V*_cmax_

Gas‐exchange measurements were taken on the largest and most accessible mature rosette leaf of stomatal development mutants and control plants at 5–7 wk post germination using a LI‐6400 Portable Photosynthesis System with the 6400‐02B LED Light Source (Li‐Cor Biosciences Inc., Lincoln, NE, USA). Gas‐exchange measurements were performed as described in Dow *et al*. (2014a,b) and the steady‐state response of net carbon assimilation (*A*) to intercellular CO_2_ concentration (*c*_i_) was obtained from stepping ambient CO_2_ at 100 ppm to 350, 500, 750 and 1000 ppm. *V*_cmax_ for each leaf was calculated by fitting individual *A*–*c*_i_ response curves to a biochemical model of C3 photosynthesis (Farquhar *et al*., 1980) using an IDL GUI computational tool developed by Bob Haxo and Joe Berry.

### Calculating *g*_smax_

Rosette leaves used in gas‐exchange experiments were prepared for stomatal phenotype analysis and quantified as described in Dow *et al*. (2014a,b). Maximum stomatal conductance to water vapor as defined by stomatal anatomy (*g*_smax_, mol H_2_O m^−2 ^s^−1^) was estimated for each leaf using a double end‐correction version of the equation by Franks & Farquhar (2001):

(Eqn 1) $$g_{s\max}=\frac{d{\mathrm{Da}}_{\max}}{v\left[1+\frac{\pi }{2}\sqrt{\frac{a_{\max}}{\pi }}\right]}$$

where *d* is the diffusivity of water in air (m^2^ s^−1^, at 22°C), *v* is the molar volume of air (m^3^ mol^−1^, at 22°C), π is the mathematical constant, approximated to 3.142, *D* is stomatal density (mm^−2^), *l* is pore depth (μm), which was equal to guard cell width at the center of the stoma, and *a*_max_ is the mean maximum stomatal pore area (μm^2^), which was defined as an ellipse with major axis equal to pore length and minor axis equal to half pore length. *g*_smax_ for each leaf was calculated as the sum of *g*_smax_ abaxial (*g*_ab_) and *g*_smax_ adaxial (*g*_ad_) using empirical values of *D*,* l* and *a*_max_ for stomata on each side of the leaf (*g*_smax_ = *g*_ab_ + *g*_ad_). *D* was determined independently for each leaf, while values of *l* and *a*_max_ were genotype averages.

### Three‐dimensional confocal imaging of leaf morphology

Imaging of the epidermis and internal leaf structures was performed using a Leica SP5 confocal microscope with the protocol developed by Wuyts *et al*. (2010). Preparation of samples was performed as described, except for the following modifications at the end of the protocol: leaves were stained by propidium iodide overnight in water and then mounted in Hoyer's solution directly on a microscope slide. Leaves were thoroughly immersed in Hoyer's solution to prevent desiccation and were left exposed to air until the leaves were transparent, at which point a cover slip was applied and imaging was performed within 48 h. Only the sixth rosette leaf was used for analysis and four areas in the midleaf region, between the midvein and leaf edge, were imaged per leaf. At each location, a *z*‐stack of images (*x*–*z* plane; see later Fig. 2a) at intervals of 2 μm was taken to span all leaf tissues, from adaxial to abaxial epidermis. Cell densities of the adaxial epidermis and palisade mesophyll were determined by hand using the Cell Counter in Fiji (NIH; www.fiji.sc/Fiji). A transvere cross‐section of the entire leaf (*x*–*z* plane, see later Fig. 2a) was produced using the Dynamic Reslice function in Fiji on a complete *z*‐stack. Leaf thickness was determined by measuring the distance betweeen the top of the adaxial epidermis and the bottom of the abaxial epidermis at three points across the image. Measurement points were visually chosen at the maximum thickness in an area, and the average of all measurements was used to define leaf thickness. Determination of leaf thickness on dehydrated samples probably underestimated the true thickness, but this error should be consistent across all samples. Leaf area was determined by outlining in pen the leaf mounted on a microscope slide, imaging the slide with a Hewlett‐Packard printer‐scanner, and calculating the area within the leaf outline using Fiji's Tracing Tool.

### Carbon isotope analysis

*Arabidopsis thaliana* seeds from a subset of genotypes were used to determine ^13^C : ^12^C isotope ratios. Although of the same genotype and lineage, seeds used in this analysis were not from the identical plants used in gas‐exchange measurements or confocal analysis of stomatal traits. Genotypes were simultaneously grown to maturity in one growth chamber, under conditions as follows: 16 : 8 h, 22 : 20°C, day : night cycle, *c*. 100 mmol photon m^−2^ s^−1^, *c*. 65% relative humidity, soil water content maintained at 70% field capacity, and ambient [CO_2_] of *c*. 425 ppm. A 2.000 mg quantity of seed was weighed and packaged in a foil ball, six replicates were performed per genotype. Samples were combusted in a Carlo Erba Combustion Elemental Analyzer (Thermo Scientific Inc., Watham, MA, USA) and the resultant gas was analyzed in a Delta V Advantage Mass Spectrometer (Thermo Scientific Inc.). The carbon isotope ratio of seed tissue (δ^13^C) in units per mil (‰) was calculated as:

(Eqn 2) $$\delta^{13}C\left(‰\right)=\left(R_{\mathrm{sample}}/R_{\mathrm{standard}}-1\right)\times 1000$$

where *R*_sample_ and *R*_standard_ are the ^13^C : ^12^C ratios of seeds and the V‐PDB standard, respectively.

### Statistical analysis

All statistical analysis was performed using R (http://www.r-project.org/). Linear regression models were used to determine variance (adjusted *R* ^2^) and the statistical significance of covariation between parameters (*P *<* *0.05). Comparison of regression models was performed by ANCOVA to determine significance between regression slopes (effect of genotype on dependent variable) or *y*‐intercepts (quantitative differences between genotypes). Comparison of mean values between Col‐0 and all other genotypes was performed by Wilcoxon signed‐rank *t*‐tests for unpaired, nonparametric samples (*P *<* *0.05).

## Results and Discussion

**Figure 1:**
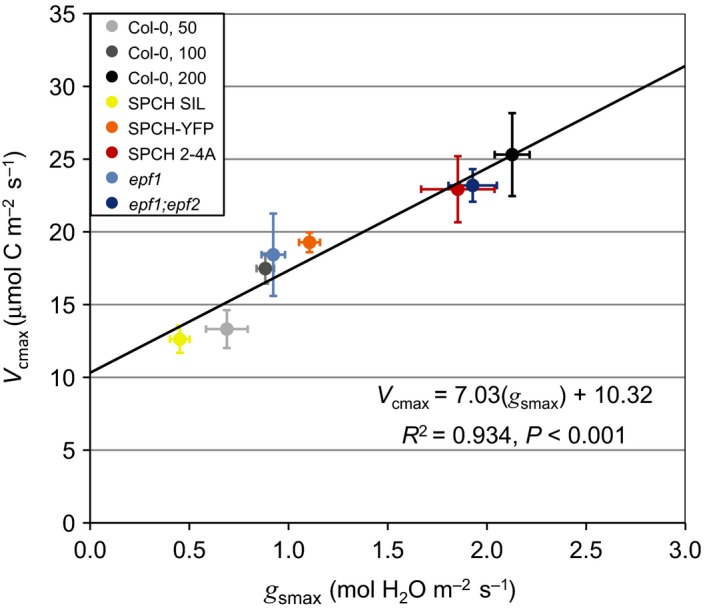
Changes in gas‐exchange capacity (*g*_smax_) driven by genetic regulators of stomatal development are correlated with changes in leaf photosynthetic potential (*V*_cmax_). *Arabidopsis thaliana* Col‐0 plants were grown at three different fluence rates (50, 100, and 200 μmol photons m^−2 ^s^−1^), while all other genotypes were grown at 100 μmol photons m^−2^ s^−1^. All individuals were grouped by genotype or light treatment and the mean values of each group were used for the regression model, which showed a positive correlation between *g*_smax_ (the maximum rate of stomatal conductance as defined by stomatal size and density) and *V*_cmax_ (the maximum rate of Rubisco carboxylation) (*R* ^2^ = 0.934, *P *<* *0.001, no. of individuals = 46). Error bars are ±SEM.

To explore the linkages between the stomatal lineage and mesophyll tissue, we analyzed carbon assimilation–intercellular CO_2_ (*A*–*c*_i_) response curves to determine *V*_cmax_, the maximum rate of carboxylation as limited by the ribulose 1,5‐bisphosphate carboxylase‐oxygenase enzyme (Rubisco). We sampled from gas‐exchange experiments of five stomatal development mutant lines and Col‐0 grown at three different fluence rates (50, 100 and 200 μmol photons m^−2^ s^−1^) as presented in Dow *et al*. (2014a,b). Indeed, there was a significant and positive relationship between *V*_cmax_ and *g*_smax_ across all lines (Fig. 1; *R* ^2^ = 0.934, *P *<* *0.001, *n* individuals = 46). While the response in *V*_cmax_ across Col‐0 light treatments was expected, we anticipated that changes in *g*_smax_ derived from genetic manipulations that target the epidermis would segregate independently from *V*_cmax_. However, our results indicate the opposite: *g*_smax_ was strongly correlated with *V*_cmax_ across a wide physiological spectrum.

*V*_cmax_ is directly related to the amount of Rubsico in the leaf (von Caemmerer & Farquhar, 1981). Our mutant lines specifically targeted the stomatal lineage, yet altering epidermal cell properties appeared to change the quantity of Rubisco in the mesophyll. If the ratio of *g*_smax_ : *V*_cmax_ remained stable across genotypes, one might predict that intrinsic water‐use efficiency (*W*_g_) should remain constant as well. As an indicator of *W*_g_ we measured carbon isotope composition (Seibt *et al*., 2008), δ^13^C, among the mutants and Col‐0 (for comparative purposes, a more negative δ^13^C indicates a lower *W*_g_). Carbon isotope measurements have previously been used to assess genetic variation in *W*_g_ because the fractionation process integrates both the diffusion and carboxylation limitations of CO_2_ uptake (Masle *et al*., 2005). Genetic controls over *W*_g_ could result from variation in stomatal properties, from modifications to the mesophyll, or from changes in both tissues. In our study, we found no significant differences in δ^13^C among mutants or transgenics despite large differences in stomatal properties (Supporting Information Table S1). This isotopic evidence further confirmed our hypothesis of interlayer developmental coordination.

**Figure 2:**
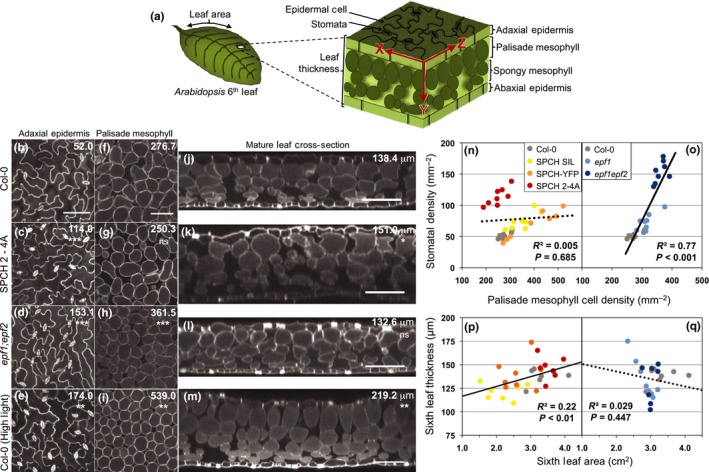
High‐resolution three‐dimensional imaging of leaf morphology reveals two separate classes of mesophyll alterations in stomatal development mutants. Images of *Arabidopsis thaliana* (b–e) depict an *x*–*z* planar view of the adaxial epidermis (see panel (a) for spatial relationships); all images of the epidermis are at the same magnification. (f–i) An *x*–*z* planar view of the palisade mesophyll cell layer; all images of the mesophyll are at the same magnification. (j–m) An *x*–*y* planar cross‐section of the mature leaf. Bars, 100 μm. The genotypes for each row of images are defined in the left margin and values in the top right corner of each image are genotype averages of stomata density (b–e), palisade cell density (f–i), or leaf thickness (j–m). Notation below the numbers indicates significant difference from Col‐0: ns, *P *>* *0.05; *, *P *<* *0.05; **, *P *<* *0.01; ***, *P *<* *0.001. All images were taken from the sixth rosette leaf (*n *=* *8 for Col‐0, SPCH 2‐4A, and *epf1;epf2*;* n *=* *4 for Col‐0 grown under high light). All SPEECHLESS (SPCH) and *epidermal patterning factor* (*epf*) lines were then grouped separately for regression analysis to determine relationships between adaxial stomatal density and palisade mesophyll cell density (n–o) and leaf thickness with leaf size (p–q). The regression analysis for each group includes the Col‐0 control plants. SPCH plants showed no significant relationship between cell type densities (n) (*R* ^2^ = 0.005, *P *=* *0.685, *n *=* *32) as indicated by the dotted line, but leaf thickness increased as a function of leaf area (p) (*R* ^2^ = 0.22, *P *<* *0.01, *n *=* *29), as indicated by the unbroken line. *epf* plants showed a positive relationship between cell type densities (o) (*R* ^2^ = 0.77, *P *<* *0.001, *n *=* *24), but leaf thickness and leaf area were not correlated (q) (*R* ^2^ = 0.029, *P *=* *0.447, *n *=* *22).

These initial findings indicated that targeted manipulations of the stomatal lineage in the epidermis were leading to concomitant changes in the underlying mesophyll tissues. Three‐dimensional confocal microscopy techniques (modified from Wuyts *et al*., 2010) allowed us to visualize the epidermis and adjacent internal structures of the leaf and therefore test this directly (Fig. 2a). We first imaged Col‐0 leaves grown under standard or high‐light conditions and focused on the relationship between the adaxial epidermis and the palisade mesophyll layer, where the majority of photosynthesis occurs (Fig. 2b–m). Plants grown in high light had increased stomatal density (Fig. 2b,e), increased palisade mesophyll density (Fig. 2f,i), and increased leaf thickness (Fig. 2j,m) relative to standard conditions, consistent with classical observations of ‘sun’ and ‘shade’ leaves across plant taxa (Nobel *et al*., 1975; Terashima *et al*., 2011). These structural changes enhance CO_2_ transfer across the epidermis and increase the internal mesophyll surface area for CO_2_ diffusion into cells, thereby enhancing photosynthetic capacity.

We then characterized interlayer coordination in mutant lines with increased stomatal production: one line expressed a hyperactive form of the SPCH transcription factor under its native promoter (active in the epidermis, SPCHp:SPCH 2‐4A) and the second was a mutant for the stomatal lineage expressed EPF peptide ligands (*epf1;epf2*). Both manipulations changed mesophyll structure (Fig. 2g,k, and 2h,l, respectively), but, unexpectedly, they changed in distinct ways. SPCH 2‐4A plants primarily displayed an increase in leaf thickness (Fig. 2j–l), while *epf1;epf2* plants increased palisade mesophyll cell density (Fig. 2f–h). Essentially, the changes in mesophyll phenotypes observed when wild‐type plants were subjected to high light were divided into discrete subresponses in these two genotypes. We tested additional related genotypes (SPCH‐YFP, SPCH SIL, and *epf1*) to explore whether these coordinate changes were specific to the respective functional classes (Fig. 2n–q). *epf* mutants had a strong positive correlation between cell type densities (*n* = 24, *R* ^2^ = 0.77, *P *<* *0.001; Fig. 2o), while multiple SPCH lines demonstrated no consistent pattern between stomatal and palisade mesophyll cell density (*n* = 32, *R* ^2^ = 0.005, *P *=* *0.685; Fig. 2n). Conversely, SPCH mutants had significant increases in leaf thickness, as well as overall leaf area (*n* = 32, *R* ^2^ = 0.22, *P *<* *0.01; Fig. 2p) in direct correlation with increasing *g*_smax_ (SPCH SIL < SPCH‐YFP < SPCH 2‐4A), while *epf* mutants had no such differences in thickness or area (*n* = 24, *R* ^2^ = 0.029, *P *=* *0.447; Fig. 2q).

The distinct effect of functional gene classes on mesophyll architecture implied that multiple mechanisms are responsible for coordination between *g*_smax_ and *V*_cmax_. Manipulating SPCH activity changed the extent of proliferation of the stomatal lineage, which increased production of nonstomatal epidermal cells that ultimately regulate leaf area (Lampard *et al*., 2008; Gonzalez *et al*., 2012). We found that leaf area was positively correlated with leaf thickness, which provided for increased cell density in three‐dimensional space and resultant increases in photosynthetic capacity (Terashima *et al*., 2006). This relationship was specific to manipulation of SPCH activity rather than manipulation of the stomatal lineage in general, as EPF mutants revealed no change in leaf area. In comparison, the change in mesophyll architecture for EPF mutants appears to be a more local event: changing the density of palisade mesophyll cells immediately below the adaxial epidermis rather than having a global effect on leaf area or thickness. Changes in mesophyll cell density were correlated with photosynthetic capacity presumably because of changes in total cell surface area, but the shape of the cells and how they pack together may also have affected surface area. Whether the local density relationship is maintained at the interface between the abaxial epidermis and the spongy mesophyll tissue was harder to determine because of the irregular spatial organization of the spongy cells and requires further investigation. To our knowledge, these results provide the first evidence that genetic changes in the stomatal lineage are linked with developmental responses in mesophyll structure. This form of interlayer communication plays a critical role in plant physiology because it appears to optimize gas exchange by matching the supply and demand for CO_2_. Our results also indicate that growth environment plays a significant role in this developmental process. For example, when wild‐type plants and EPF mutants are grown under full sunlight, differences in *V*_cmax_ are insignificant while changes in *g*_smax_ driven by EPF activity remain (Franks *et al*., 2015). Growth under high‐ or low‐intensity light could also help to explain the observed differences in *W*_g_ as measured by carbon isotope composition across these different studies (Franks *et al*., 2015).

**Figure 3:**
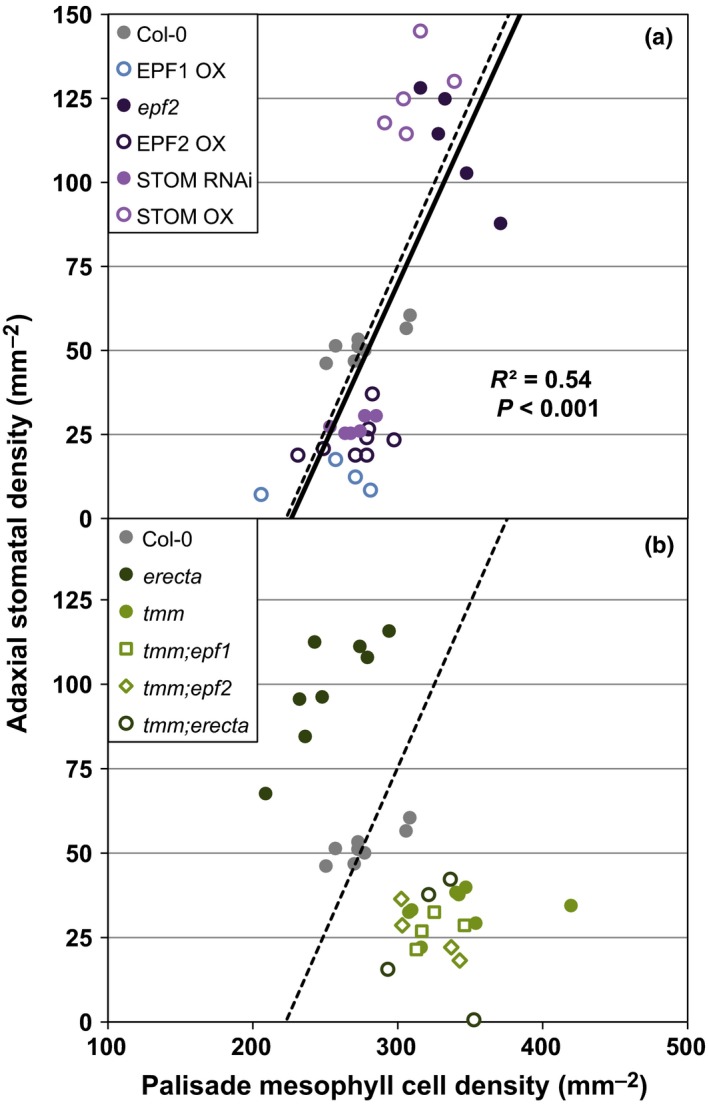
Cell surface receptors, but not ligands, involved in stomatal development can disrupt the coordination between epidermal and mesophyll cell densities. In panel (a), manipulation of various EPIDERMAL PATTERNING FACTORS (EPFs) in *Arabidopsis thaliana* mirrors the epidermal–mesophyll relationship shown in Fig. 2(o) (solid line, *R* ^2^ = 0.54, *P *<* *0.001, *n *=* *35). The dashed lines in (a, b) refer to the regression model from Fig. 2(o); comparison of regression models was not significantly different in (a) (ANCOVA, *P *=* *0.896). In (b), loss of cell surface receptors TOO MANY MOUTHS (TMM) and ERECTA altered the relationship between adaxial stomatal density and palisade mesophyll cell density in opposite ways. *erecta* plants increased adaxial stomata but decreased mesophyll cell density, while *tmm* decreased adaxial stomata and increased mesophyll cell density (see also Supporting Information Table S2). *tmm* was epistatic in all double mutant combinations tested (see also Table S3).

Despite these environmental influences, this type of developmental linkage provides a blueprint for harnessing stomatal development to alter biochemical capacity in the mesophyll. One stomatal regulator that can strongly influence *W*_g_ is ERECTA (Masle *et al*., 2005), a receptor‐like kinase that mediates EPF signaling (Lee *et al*., 2012, 2015). ERECTA was demonstrated to influence both the proliferation of stomata in the epidermis and mesophyll cell development (Masle *et al*., 2005). In light of our work, this dual role appears to be a critical link for improving *W*_g_. To pursue a potential mechanism, we sampled mutants of the cell surface receptor genes, *ERECTA* and *TMM*, and additional lines of the *EPF* family, including *STOMAGEN*, an antagonist of EPF1 and EPF2 activity that travels from the mesophyll to the epidermis (Sugano *et al*., 2009; Hunt *et al*., 2010). Loss of function or overexpression of STOMAGEN was consistent with similar manipulations of EPF1 and EPF2, in that stomatal density and palisade cell density remained positively correlated (*n* = 35, *R* ^2^ = 0.54, *P *<* *0.001; Fig. 3a). While each of the EPF family members affected the overall ‘set point’ of epidermal and mesophyll cell numbers, all lines remained consistent with respect to interlayer coordination, thus ruling them out as the required signal. By contrast, *erecta* and *tmm* had quantifiably opposite effects on the density of stomata and palisade mesophyll cells. *erecta* increased adaxial stomatal density and trended toward decreased palisade mesophyll density relative to Col‐0 (*P *<* *0.001 and *P *=* *0.128, respectively; Fig. 3b and Table S2). *tmm* had the reverse effect – a decrease in adaxial stomata but an increase in palisade mesophyll density (*P *<* *0.001 and *P *<* *0.01, respectively; Fig. 3b and Table S2). Essentially, mutations in these coreceptors broke the developmental coordination between epidermal and mesophyll tissues.

This conclusion was further corroborated by carbon isotope analysis, as *tmm* had a −2.1‰ difference relative to Col‐0 (δ^13^C of −31.8‰ and −33.9‰, respectively, *P *<* *0.01), which implies an improvement in *W*_g_. *ERECTA* mutants exhibited the opposite trend, consistent with the published difference of +1.1‰ (Masle *et al*., 2005). The change in δ^13^C for *tmm* was not the result of changes in stomatal conductance owing to stomatal clustering, as a control line for clustering (*basl*; characterized in Table S2 and Dow *et al*., 2014a,b) was no different from Col‐0 (−33.9‰ and −33.9‰, respectively, *P *=* *0.675). These isotopic results imply that *TMM* can negatively regulate *W*_g_, which directly antagonizes the previously identified role of *ERECTA* (Masle *et al*., 2005). While the *erecta* mutant has multiple effects on plant architecture resulting from widespread expression patterns, *ERECTA*'s genetic relationship with *TMM* and their opposing effects on δ^13^C indicate that coordination of leaf epidermal and mesophyll development is instrumental in the determination of *W*_g_. In addition, the epidermal‐specific TMM appears to be the critical mediator of the interlayer signaling process: loss of *TMM* was epistatic to loss of the *EPF* ligands, and, most interestingly, it was epistatic to loss of its coreceptor *ERECTA* (Fig. 3b; Table S3). Both synergistic and antagonistic relationships between *ERECTA* and *TMM* have been described for developmental responses within the stomatal lineage (Shpak *et al*., 2005; Qi *et al*., 2017). How these coreceptors manifest opposite effects on interlayer coordination remains unknown: the TMM‐ERECTA receptor complex may receive and propagate a direct signal to coordinate interlayer development or it might act through some secondary and more indirect mechanism. Regardless, the capacity to disrupt interlayer coordination indicates that this process is genetically defined over developmental timescales and relies upon signaling in the epidermis. This distinguishes interlayer coordination from being a strictly homeostatic mechanism responding to changes in stomatal conductance, photosynthesis, leaf water status, sugar content, or another indicator of leaf physiology.

Our study reinforces the importance of developmental coordination between leaf tissues and identifies a multifaceted process in leaves that appears dependent on both local communication and tissue‐wide responses to align gas‐exchange potential with photosynthetic capacity. While the full mechanism driving coordination remains unknown, elements of the developmental process can be separated into at least two subcategories: cellular density and leaf thickness. The former process, observed here as parallel changes in palisade mesophyll density and adaxial stomatal density, requires TMM signaling, potentially acting in opposition to its coreceptor ERECTA. Understanding how TMM and ERECTA drive changes in leaf development across tissue layers is a potentially rewarding avenue for improving plant water‐use efficiency. Independently altering the epidermal or mesophyll developmental process, or driving opposing responses, may reduce transpiration without compromising photosynthesis or plant growth. This approach also highlights an important caveat for genetic engineering of agricultural or bioenergy feedstocks: our efforts must consider the compensatory changes that are induced when manipulating plant anatomy and function. With respect to water‐use efficiency, the developmental linkage between *g*_smax_ and *V*_cmax_ may be a valuable tool for measuring noncompensatory improvements in plant productivity and responses to key environmental parameters, such as increasing atmospheric CO_2_ (Leakey *et al*., 2009; Lammertsma *et al*., 2011).

## Author contributions

G.J.D., J.A.B., and D.C.B. designed the project, interpreted the data, and wrote the manuscript; G.J.D. performed the experiments.

## Supporting information

Please note: Wiley Blackwell are not responsible for the content or functionality of any Supporting Information supplied by the authors. Any queries (other than missing material) should be directed to the *New Phytologist* Central Office.

**Table S1** Summary of gas‐exchange (*g*_smax_) and photosynthetic (*V*_cmax_) capacity among stomatal development mutants and the impact on water‐use efficiency (δ^13^C)
**Table S2** Summary of cell anatomical features and leaf attributes for all controls and genotypes visualized using three‐dimensional confocal microscopy
**Table S3** Test of epistasis among signaling genotypes and double mutants using stomatal density (SD) to palisade mesophyll density (PMD) ratio

## References

[nph14746-bib-0001] Ball J , Woodrow I , Berry J . 1987 A model predicting stomatal conductance and its contribution to the control of photosynthesis under different environmental conditions In: Biggens J, ed. Prog. Photosynthesis Res. Proc. Int. Congress 7th, Providence; 10–15 Aug 1986, *vol 4* Boston, MA, USA: Kluwer: 221–224.

[nph14746-bib-0002] von Caemmerer S , Farquhar GD . 1981 Some relationships between the biochemistry of photosynthesis and the gas exchange of leaves. Planta 153: 376–387. 24276943 10.1007/BF00384257

[nph14746-bib-0003] Chater CCC , Oliver J , Casson S , Gray JE . 2014 Putting the brakes on: abscisic acid as a central environmental regulator of stomatal development. New Phytologist 202: 376–391. 24611444 10.1111/nph.12713

[nph14746-bib-0004] Doheny‐Adams T , Hunt L , Franks PJ , Beerling DJ , Gray JE . 2012 Genetic manipulation of stomatal density influences stomatal size, plant growth and tolerance to restricted water supply across a growth carbon dioxide gradient. Philosophical Transactions of the Royal Society B: Biological Sciences 367: 547–555. 10.1098/rstb.2011.0272 PMC3248714 22232766

[nph14746-bib-0005] Dong J , MacAlister CA , Bergmann DC . 2009 BASL controls asymmetric cell division in *Arabidopsis* . Cell 137: 1320–1330. 19523675 10.1016/j.cell.2009.04.018 PMC4105981

[nph14746-bib-0006] Dow GJ , Bergmann DC . 2014 Patterning and processes: how stomatal development defines physiological potential. Current Opinion in Plant Biology 21C: 67–74. 10.1016/j.pbi.2014.06.007 25058395

[nph14746-bib-0007] Dow GJ , Bergmann DC , Berry JA . 2014a An integrated model of stomatal development and leaf physiology. New Phytologist 201: 1218–1226. 24251982 10.1111/nph.12608

[nph14746-bib-0008] Dow GJ , Berry JA , Bergmann DC . 2014b The physiological importance of developmental mechanisms that enforce proper stomatal spacing in *Arabidopsis thaliana* . New Phytologist 201: 1205–1217. 24206523 10.1111/nph.12586

[nph14746-bib-0009] Farquhar GD , von Caemmerer S , Berry JA . 1980 A biochemical model of photosynthetic CO_2_ assimilation in leaves of C_3_ species. Planta 149: 78–90. 24306196 10.1007/BF00386231

[nph14746-bib-0010] Franks PJ , Doheny‐Adams TW , Britton‐Harper ZJ , Gray JE . 2015 Increasing water‐use efficiency directly through genetic manipulation of stomatal density. New Phytologist 201: 188–195. 10.1111/nph.13347 25754246

[nph14746-bib-0011] Franks PJ , Farquhar GD . 2001 The effect of exogenous abscisic acid on stomatal development, stomatal mechanics, and leaf gas exchange in *Tradescantia virginiana* . Plant Physiology 125: 935–942. 11161050 10.1104/pp.125.2.935 PMC64894

[nph14746-bib-0012] Gonzalez N , Vanhaeren H , Inze D . 2012 Leaf size control: complex coordination of cell division and expansion. Trends in Plant Science 17: 332–340. 22401845 10.1016/j.tplants.2012.02.003

[nph14746-bib-0013] Hara K , Kajita R , Torii KU , Bergmann DC , Kakimoto T . 2007 The secretory peptide gene EPF1 enforces the stomatal one‐cell‐spacing rule. Genes & Development 21: 1720–1725. 17639078 10.1101/gad.1550707 PMC1920166

[nph14746-bib-0014] Hara K , Yokoo T , Kajita R , Onishi T , Yahata S , Peterson KM , Torii KU , Kakimoto T . 2009 Epidermal cell density is autoregulated via a secretory peptide, EPIDERMAL PATTERNING FACTOR 2 in *Arabidopsis* leaves. Plant and Cell Physiology 50: 1019–1031. 19435754 10.1093/pcp/pcp068

[nph14746-bib-0015] Hunt L , Bailey KJ , Gray JE . 2010 The signalling peptide EPFL9 is a positive regulator of stomatal development. New Phytologist 186: 609–614. 20149115 10.1111/j.1469-8137.2010.03200.x

[nph14746-bib-0016] Hunt L , Gray JE . 2009 The signaling peptide EPF2 controls asymmetric cell divisions during stomatal development. Current Biology 19: 864–869. 19398336 10.1016/j.cub.2009.03.069

[nph14746-bib-0017] Lammertsma EI , de Boer HJ , Dekker SC , Dilcher DL , Lotter AF , Wagner‐Cremer F . 2011 Global CO_2_ rise leads to reduced maximum stomatal conductance in Florida vegetation. Proceedings of the National Academy of Sciences, USA 108: 4035–4040. 10.1073/pnas.1100371108 PMC3054011 21330552

[nph14746-bib-0018] Lampard GR , MacAlister CA , Bergmann DC . 2008 *Arabidopsis* stomatal initiation is controlled by MAPK‐mediated regulation of the bHLH SPEECHLESS. Science 322: 1113–1116. 19008449 10.1126/science.1162263

[nph14746-bib-0019] Lau OS , Bergmann DC . 2012 Stomatal development: a plant's perspective on cell polarity, cell fate transitions and intercellular communication. Development 139: 3683–3692. 22991435 10.1242/dev.080523 PMC3445305

[nph14746-bib-0020] Lawson T , Blatt MR . 2014 Stomatal size, speed, and responsiveness impact on photosynthesis and water use efficiency. Plant Physiology 164: 1556–1570. 24578506 10.1104/pp.114.237107 PMC3982722

[nph14746-bib-0021] Leakey ADB , Ainsworth EA , Bernacchi CJ , Rogers A , Long SP , Ort DR . 2009 Elevated CO_2_ effects on plant carbon, nitrogen, and water relations: six important lessons from FACE. Journal of Experimental Botany 60: 2859–2876. 19401412 10.1093/jxb/erp096

[nph14746-bib-0022] Lee JS , Hnilova M , Maes M , Lin Y‐CL , Putarjunan A , Han S‐K , Avila J , Torii KU . 2015 Competitive binding of antagonistic peptides fine‐tunes stomatal patterning. Nature 522: 439–443. 26083750 10.1038/nature14561 PMC4532310

[nph14746-bib-0023] Lee JS , Kuroha T , Hnilova M , Khatayevich D , Kanaoka MM , McAbee JM , Sarikaya M , Tamerler C , Torii KU . 2012 Direct interaction of ligand‐receptor pairs specifying stomatal patterning. Genes & Development 26: 126–136. 22241782 10.1101/gad.179895.111 PMC3273837

[nph14746-bib-0024] Masle J , Gilmore SR , Farquhar GD . 2005 The ERECTA gene regulates plant transpiration efficiency in *Arabidopsis* . Nature 436: 866–870. 16007076 10.1038/nature03835

[nph14746-bib-0025] Nadeau JA , Sack FD . 2002 Control of stomatal distribution on the *Arabidopsis* leaf surface. Science 296: 1697–1700. 12040198 10.1126/science.1069596

[nph14746-bib-0026] Nobel PS , Zaragoza LJ , Smith WK . 1975 Relation between mesophyll surface area, photosynthetic rate, and illumination level during development for leaves of *Plectranthus parviflorus* Henckel. Plant Physiology 55: 1067–1070. 16659211 10.1104/pp.55.6.1067 PMC541767

[nph14746-bib-0027] Pillitteri LJ , Torii KU . 2012 Mechanisms of stomatal development. Annual Review of Plant Biology 63: 591–614. 10.1146/annurev-arplant-042811-105451 22404473

[nph14746-bib-0028] Qi X , Han S‐K , Dang JH , Garrick JM , Ito M , Hofstetter AK , Torii KU . 2017 Autocrine regulation of stomatal differentiation potential by EPF1 and ERECTA‐LIKE1 ligand‐receptor signaling. eLife 6: e24102. 28266915 10.7554/eLife.24102 PMC5358980

[nph14746-bib-0029] Seibt U , Rajabi A , Griffiths H , Berry JA . 2008 Carbon isotopes and water use efficiency: sense and sensitivity. Oecologia 155: 441–454. 18224341 10.1007/s00442-007-0932-7

[nph14746-bib-0030] Shpak ED , McAbee JM , Pillitteri LJ , Torii KU . 2005 Stomatal patterning and differentiation by synergistic interactions of receptor kinases. Science 309: 290–293. 16002616 10.1126/science.1109710

[nph14746-bib-0031] Sugano S , Shimada T , Imai Y , Okawa K , Tamai A , Mori M , Hara‐Nishimura I . 2009 Stomagen positively regulates stomatal density in *Arabidopsis* . Nature 463: 241–244. 20010603 10.1038/nature08682

[nph14746-bib-0032] Tanaka Y , Sugano SS , Shimada T , Hara‐Nishimura I . 2013 Enhancement of leaf photosynthetic capacity through increased stomatal density in *Arabidopsis* . New Phytologist 198: 757–764. 23432385 10.1111/nph.12186

[nph14746-bib-0033] Terashima I , Hanba YT , Tazoe Y , Vyas P , Yano S . 2006 Irradiance and phenotype: comparative eco‐development of sun and shade leaves in relation to photosynthetic CO_2_ diffusion. Journal of Experimental Botany 57: 343–354. 16356943 10.1093/jxb/erj014

[nph14746-bib-0034] Terashima I , Hanba YT , Tholen D , Niinemets U . 2011 Leaf functional anatomy in relation to photosynthesis. Plant Physiology 155: 108–116. 21075960 10.1104/pp.110.165472 PMC3075775

[nph14746-bib-0035] Torii KU , Mitsukawa N , Oosumi T , Matsuura Y , Yokoyama R , Whittier RF , Komeda Y . 1996 The *Arabidopsis* ERECTA gene encodes a putative receptor protein kinase with extracellular leucine‐rich repeats. Plant Cell 8: 735–746. 8624444 10.1105/tpc.8.4.735 PMC161133

[nph14746-bib-0036] Wuyts N , Palauqui JC , Conejero G , Verdeil JL , Granier C , Massonnet C . 2010 High‐contrast three‐dimensional imaging of the *Arabidopsis* leaf enables the analysis of cell dimensions in the epidermis and mesophyll. Plant Methods 6: 17. 20598116 10.1186/1746-4811-6-17 PMC2909956

