# Supplementary material for: Disruption of stomatal lineage signaling or transcriptional regulators has differential effects on mesophyll development, but maintains coordination of gas exchange
Source: New Phytol. 2017 Aug 21;216(1):69–75. doi: 10.1111/nph.14746 (PMC5601202; doi:10.1111/nph.14746)
Supplement: Supplementary file 1 — Table S1 Summary of gas‐exchange (g smax) and photosynthetic (V cmax) capacity among stomatal development mutants and the impact on water‐use efficiency (δ13C) Table S2 Summary of cell anatomical features and leaf attributes for all controls and genotypes visualized using three‐dimensional confocal microscopy Table S3 Test of epistasis among signaling genotypes and double mutants using stomatal density (SD) to palisade mesophyll density (PMD) ratio [file NPH-216-69-s001.pdf]

New Phytologist Supporting Information Tables S1-S3

Article Title: Disruption of stomatal lineage signaling or transcriptional regulators has differential affects on mesophyll development, but maintains coordination of gas-exchange

Authors: Graham J. Dow, Joseph A. Berry, Dominique C. Bergmann

Article Acceptance Date: July 14, 2017

Genotype (growth condition)[1]	g_{smax} (mol H_2O $m^{-2}s^{-1}$)	V_{cmax} (µmol C $m^{-2}s^{-1}$)	Carbon Isotope Ratio ($\delta^{13}C$, ‰)[2]
SPCH SIL	0.452 ± 0.050	12.62 ± 0.94	-33.7 ± 0.04
Col-0 (low light: 50)	0.689 ± 0.105	13.32 ± 1.30	--
Col-0	0.883 ± 0.044	17.48 ± 1.04	-33.9 ± 0.05
epf1	0.923 ± 0.059	18.43 ± 2.83	-34.0 ± 0.02
SPCH-YFP	1.106 ± 0.053	19.28 ± 0.68	-33.8 ± 0.04
SPCH 2-4A	1.853 ± 0.185	22.93 ± 2.27	-34.0 ± 0.06
epf1;epf2	1.928 ± 0.122	23.19 ± 1.12	-33.8 ± 0.08
Col-0 (high light: 200)	2.127 ± 0.088	25.31 ± 2.85	--

Table S1. Summary of gas-exchange (g_{smax}) and photosynthetic (V_{cmax}) capacity among stomatal development mutants and the impact on water-use efficiency ($\delta^{13}C$). All values are mean ± SEM, $4<n<8$ per genotype; n=6 per genotype for carbon isotope measurements, $\delta^{13}C$, which was used to infer intrinsic water-use efficiency (W_g). [1]Light conditions for growth were 100 µmol photons $m^{-2}s^{-1}$, unless noted otherwise. [2]No significant differences in $\delta^{13}C$ (P>0.05) were observed between Col-0 and any other genotypes.

Genotype (Light Intensity)	Adaxial Stomatal Density (mm^{-2})	Adaxial Epidermal Density (mm^{-2})	Adaxial Stomatal Index	Palisade Mesophyll Density (mm^{-2})	Leaf Thickness (μm)	Sixth Leaf Area (cm^2)	Number of Plants (*n*)
Col-0	52.0 ± 1.7	169.2 ± 4.9	0.235 ± 0.003	276.7 ± 7.3	138.4 ± 2.7	3.34 ± 0.15	8
Col-0 (Low)	57.6 ± 3.8	261.0 ± 10.9	0.179 ± 0.005	430.4 ± 19.7	86.1 ± 3.6	1.52 ± 0.17	4
Col-0 (High)	174.0 ± 4.7	356.1 ± 8.1	0.328 ± 0.002	539.0 ± 12.0	219.2 ± 6.1	2.51 ± 0.10	4
SPCH SIL	74.7 ± 3.0	366.8 ± 10.6	0.169 ± 0.007	351.3 ± 9.6	124.4 ± 4.1	2.19 ± 0.18	8
SPCH-YFP	69.8 ± 3.6	236.7 ± 9.3	0.224 ± 0.006	394.6 ± 15.8	139.5 ± 5.9	2.65 ± 0.16	8
SPCH 2-4A	114.0 ± 2.8	322.1 ± 9.3	0.262 ± 0.004	250.3 ± 7.0	151.0 ± 5.8	3.52 ± 0.09	8
epf1	74.0 ± 4.6	171.0 ± 7.0	0.300 ± 0.006	315.9 ± 9.8	134.9 ± 7.3	2.84 ± 0.10	8
EPF1 OX	11.4 ± 1.7	307.3 ± 9.4	0.035 ± 0.003	253.5 ± 9.4	139.1 ± 7.5	3.44 ± 0.36	4
epf2	111.6 ± 7.4	308.3 ± 16.0	0.265 ± 0.004	338.7 ± 9.5	115.0 ± 4.2	2.96 ± 0.20	5
EPF2 OX	23.6 ± 2.2	147.0 ± 3.6	0.136 ± 0.008	270.7 ± 7.5	133.9 ± 8.9	3.24 ± 0.19	8
epf1;epf2	153.1 ± 3.6	253.5 ± 7.4	0.378 ± 0.004	361.5 ± 6.3	132.6 ± 6.9	3.02 ± 0.06	8
STOMAGEN RNAi	27.5 ± 1.1	155.8 ± 3.4	0.150 ± 0.004	269.9 ± 5.0	135.5 ± 7.9	3.25 ± 0.25	6
STOMAGEN OX	126.4 ± 4.3	216.2 ± 5.5	0.368 ± 0.005	310.9 ± 6.7	130.9 ± 4.8	2.90 ± 0.10	5
erecta	98.9 ± 2.4	298.3 ± 8.3	0.250 ± 0.004	251.8 ± 5.5	145.7 ± 5.1	3.02 ± 0.23	8
tmm	33.4 ± 1.7	182.6 ± 4.2	0.153 ± 0.007	342.0 ± 7.4	137.5 ± 3.3	2.76 ± 0.17	8
tmm;erecta	23.1 ± 5.4	231.4 ± 7.2	0.082 ± 0.018	333.6 ± 9.1	125.7 ± 3.0	2.90 ± 0.27	4
tmm;epf1	27.4 ± 2.7	170.7 ± 2.6	0.135 ± 0.009	325.7 ± 7.0	129.6 ± 3.1	3.18 ± 0.11	4
tmm;epf2	26.3 ± 2.5	173.0 ± 3.8	0.129 ± 0.009	321.3 ± 6.8	136.8 ± 3.8	3.70 ± 0.06	4
basl	91.7 ± 4.3	184.4 ± 9.9	0.333 ± 0.005	291.2 ± 8.2	147.2 ± 4.1	3.00 ± 0.18	4

Table S2. Summary of cell anatomical features and leaf attributes for all controls and genotypes visualized using three-dimensional confocal microscopy. All values are mean ± SEM.

Genotype	SD:PMD Ratio	Comparison to *tmm*
tmm	0.098 ± 0.006	--
erecta	0.392 ± 0.014	p < 0.001 ***
epf1	0.233 ± 0.010	p < 0.001 ***
epf2	0.333 ± 0.030	p < 0.01 **
tmm;erecta	0.075 ± 0.043	p = 1.00 ns
tmm;epf1	0.084 ± 0.007	p = 0.2337 ns
tmm;epf2	0.083 ± 0.017	p = 0.4606 ns

Table S3: Test of epistasis among signaling genotypes and double mutants using stomatal density (SD) to palisade mesophyll density (PMD) ratio. All values are mean ± SEM. Notation in third column indicates significant difference from *tmm*, where not significant (ns) when $P > 0.05$, * when $P < 0.05$, ** when $P < 0.01$, and *** when $P < 0.001$.
